# Evaluation of the efficacy of dasatinib, a Src/Abl inhibitor, in colorectal cancer cell lines and explant mouse model

**DOI:** 10.1371/journal.pone.0187173

**Published:** 2017-11-01

**Authors:** Aaron J. Scott, Eun-Kee Song, Stacey Bagby, Alicia Purkey, Martin McCarter, Csaba Gajdos, Kevin S. Quackenbush, Benjamin Cross, Todd M. Pitts, Aik Choon Tan, S. Gail Eckhardt, Hubert Fenton, John Arcaroli, Wells A. Messersmith

**Affiliations:** 1 Division of Medical Oncology, Banner University of Arizona Cancer Center, Tucson, AZ, United States of America; 2 Chonbuk National University Medical School, Jeonju, South Korea; 3 Division of Medical Oncology, University of Colorado Denver Anschutz Medical Campus and University of Colorado Cancer Center, Aurora, CO, United States of America; 4 Department of Surgery, University of Colorado Denver Anschutz Medical Campus and University of Colorado Cancer Center, Aurora, CO, United States of America; 5 Division of Medical Oncology, The University of Texas at Austin, Austin, TX, United States of America; 6 Department of Pathology, University of Colorado Denver Anschutz Medical Campus and University of Colorado Cancer Center, Aurora, CO, United States of America; University of South Alabama Mitchell Cancer Institute, UNITED STATES

## Abstract

**Background:**

Dysregulation of the Src pathway has been shown to be important at various stages of cancer. Dasatinib is a potent Src/Abl inhibitor and has demonstrated to have anti-proliferative and anti-invasive activity in many preclinical models. The objective of this study was to determine the anti-tumor activity of dasatinib using *in vitro* and *in vivo* preclinical colorectal (CRC) models.

**Methods:**

CRC cell lines and patient-derived tumor explant (PDX) models were used to investigate the efficacy of dasatinib. We treated 50 CRC cell lines with dasatinib for 72 hours and proliferation was assayed by a sulforhodamine B (SRB) assay; an IC_50_ ≤ 0.08 μmol/L was considered sensitive. We treated 17 patient-derived CRC explants with dasatinib (50 mg/kg/day, administered once-daily) for 28 days to determine *in vivo* efficacy. Tumor growth inhibition (TGI) ≥ 50% was considered sensitive.

**Results:**

We found that 8 out of 50 CRC cell lines reached an IC_50_ ≤ 0.08 μmol/L with dasatinib treatment. In addition, of 17 CRC explants grown in the xenograft mouse model, 2 showed sensitivity to dasatinib. The anti-tumor effects observed in this study were a result of G1 cell cycle arrest as the dasatinib sensitive CRC cell lines exhibited G1 inhibition. Moreover, those CRC cell lines that were responsive (0.08 μmol/L) to treatment demonstrated a significant baseline increase in Src and FAK gene expression.

**Conclusion:**

Dasatinib demonstrated significant anti-proliferative activity in a subset of CRC cell lines *in vitro*, especially in those with increased Src expression at baseline, but only showed modest efficacy in CRC explants. Dasatinib is currently being studied in combination with chemotherapy in patients with advanced CRC, as its use as a single agent appears limited.

## Introduction

Colorectal cancer (CRC) is one of the most common malignancies in Western countries, and the prognosis remains dismal in the metastatic setting [1]. Approximately 50% of patients with advanced colorectal cancer will ultimately die from their disease, and the 5-year survival is approximately 11% in the metastatic setting [2]. Src, a pleiotropic nonreceptor tyrosine kinase, is an intracellular protein that modulates important signal transducers. Aberrant Src activity has been shown to play a central role in tumorigenesis of all stages of CRC [3, 4]. The Src pathway is activated in approximately 80% of all human colon tumors, including adenomatous polyps, and this activation is an important factor in the induction and progression of tumors [5]. Talamonti *et al*. demonstrated that Src activity was greatest in liver metastatic lesions, supporting the theory of the importance of Src activation and cancer progression [6]. Clinically, Src activation is an independent prognostic indicator at all stages of CRC, further demonstrating its vital role in sequential progression of pre-malignant tumors, to malignancy, and ultimately tumor metastasis [7].

Physiologic activity of Src includes participation in cell-cell adhesion regulation via modulation of integrins and fibroblast proliferation via activation of focal adhesion kinase (FAK) and STAT-3, a protein important in cell proliferation and survival. In addition, Src interacts in a complex and bidirectional way with multiple receptor tyrosine kinases (RTKs) that are important for cell proliferation and migration, including epidermal growth factor receptor (EGFR), human epidermal growth factor receptor 2/Neu (HER2/Neu), platelet-derived growth factor receptor (PDGFR), fibroblast growth factor receptor (FGFR), hepatocyte growth factor (HGF) receptor, colony stimulating factor-1 receptor (CSF-1R), insulin-like growth factor-1 receptor (IGF-1R), and stem cell factor receptor (c-Kit) [8–16]. Overregulation or pathologic expression of Src consequently leads to cancer progression via multiple signal transduction pathways. This has led to the development of multiple agents aimed at inhibiting Src as a therapeutic method for treating solid tumors.

Dasatinib (Sprycel®) is an ATP-competitive inhibitor of BCR-ABL, ephrin (EPH), c-Kit, PDGFRβ, and Src family kinases and has been approved by the United States Food and Drug Administration (FDA) for the treatment of adult chronic myeloid leukemia (CML) and Ph+ acute lymphoblastic leukemia (ALL) [17]. Preclinical studies have shown some efficacy with dasatinib in multiple tumor types including CRC [18]. In addition, the combination of dasatinib and oxaliplatin appear to be additive in the treatment of CRC cell lines [19]. In a restrospective analysis done by Kopetz *et al*., patients that received neoadjuvant oxaliplatin prior to their hepatectomy for metastatic colorectal cancer had higher levels of Src pathway signalling and poorer relapse-free survival compared with those who did not receive oxaliplatin, suggesting that combination therapy with Src inhibition and chemotherapy may be more efficacious than chemotherapy alone [20]. These studies demonstrate that dasatinib may be efficacious in patients with CRC and warrants further exploration in the treatment of colorectal cancer. This manuscript describes the investigation of dasatinib in CRC cell lines and patient-derived tumor xenograft mouse models.

## Materials and methods

### CRC xenograft model

Colorectal cancer patient-derived tumor specimens were acquired from consenting patients at the University of Colorado Hospital in accordance with protocols approved by the Colorado Multiple Institutional Review Board. Female athymic nude (nu+/nu+) mice (6–8 weeks of age) were purchased from Envigo under an approved research protocol by the Institutional Animal Care and Use Committee (IUCAC). The expansion of tumor specimens for treatment studies were performed as previously described [21]. Briefly, tumors were expanded in the left and right flanks of 5–6 mice (10 evaluable tumors per group). Mice were euthanized if the tumor reached 2000mm^3^ in any direction or weight loss was greater than 15% as per IACUC protocol. Mice were randomized into vehicle or dasatinib groups when tumor volumes reached ~200 mm^3^. Mice were treated daily with dasatinib (50 mg/kg- daily x 5 days) for 28 days. Mice were monitored daily for signs of toxicity and tumor size was evaluated twice per week by calliper measurements using the following formula: tumor volume = [length x width^2^] * 0.52. Animals were housed 5 per cage in ventilated cages and allowed free access to food and water.

### CRC cell lines and culture

The CRC cell lines examined in this study were purchased from the American Type Culture Collection (ATCC), European Collection of Cell Cultures (ECACC) and Korean Cell Line Bank (KCLB)—Seoul National University. Cells were maintained and treated in RPMI media that contained 10% fetal bovine serum, 1% nonessential amino acids, and 1% penicillin/streptomycin. The cells were placed in an incubator at 37°C containing 5% CO_2_. The cells were routinely screened for the presence of Mycoplasma (MycoAlert; Cambrex BioScience). The CRC cell lines were treated with dasatinib when they reached ∼70% confluence. All cell lines were tested and authenticated in the University of Colorado Cancer Center DNA Sequencing and Analysis Core. CRC cell line DNA was tested using the Profiler Plus kit (Applied Biosystems). The data obtained were compared with cell line databases to ensure the cell lines have not changed.

### Anti-proliferative assessment

The anti-proliferative effects of dasatinib were determined using a sulforhodamine B (SRB) assay (13). Cells in logarithmic growth phase were plated into 96-well cell culture treated plates. Cells were plated at a concentration 500–3,000 viable cells in 100 ul of complete media per well and incubated overnight before treated to varying concentrations of dasatinib (5, 2.5, 1.25, 0.625, 0.32, 0.16, and 0.08 μmol/L) for 72 hours. After 72 hours of exposure to dasatinib, 50 ul of cold 10% TCA was added to the well after media removal and the cells were incubated for 30 minutes at 4°C. Following fixation, cells were then washed with water and stained with 0.4% sulforhodamine B for 30 minutes at room temperature. The plates were washed with 1% acetic acid followed by stain solubilization with 10 mM Tris. A synergy 2 plate reader (Biotek) was used to determine the effects of treatment on the CRC cell lines.

### Cell cycle analysis and apoptosis evaluation

For treatment effects on cell cycle, the sensitive (HCT116 and LS174T) and resistant (HT15 and SW620) CRC cell lines (2 × 10^5^ per well) were plated in six-well plates. The next day, dasatinib (0.08 μmol/L) was added to the wells and incubated for 24 hours. After 24 hours of treatment the wells were washed in PBS, and Krishanaposs stain was added to each well. Flow cytometry analysis (University of Colorado Cancer Center Flow Cytometry Core Facility) was performed on the cells after 24 hours of staining. For determination of the treatment effects on apoptosis, the sensitive and resistant CRC cell lines were plated in a 96-well white-walled plate. The cells were then treated with dasatinib (0.08 μmol/L) for 24 hours and apoptosis was determined by the measurement of caspase-3/7 activity using a luminometric Caspase-Glo-3/7 assay (Promega) according to the manufacturer's protocol and read using a 96-well plate reader (Biotek).

### Immunoblotting

Dasatinib treated CRC cell lines (con, 0.5h, 1h, 2h, 4h, and 8h) and tumor (end of study) protein concentration were determined using the 660 Protein Assay kit. Fifty micrograms of sample were run on 4–12% Bis-Tris precast gels (Life Technologies, Carlsbad, CA). Following electrophoresis, the proteins were transferred to a nitrocellulose membrane using an iBlot (Life Technologies, Carlsbad, CA). The membranes were blocked at room temperature with 5% nonfat milk (BioRad) in TBS/tween 20 (TBST) for 1 hour at room temperature. The following primary antibodies were used at a concentration of 1:1000: p/t Src, p/t FAK, p/t paxillin, p/t AKT, p/t Stat3, and Actin (Cell Signaling, Danvers, MA) and the membranes were incubated overnight at 4°C with rocking. The membranes were washed three times with TBST, and anti-rabbit or anti-mouse IgG horseradish peroxidase–conjugated antibody were added at a final dilution of 1:50,000 in TBST. After washing three times with TBST, bound antibodies were detected by enhanced chemiluminescence (Millipore, ‎Billerica, MA).

### Gene pathway analysis by RNA Seq

Total RNA from CRC explants were extracted using RNAeasy kit (Qiagen) and profiled using Affymetrix gene array and RNA Seq. Raw expression values were extracted and normalized by the Affymetrix Power Tools based on robust multiarray average approach. Multiple probe sets representing the same gene were collapsed by the maximum value. To analyze the pathway enriched in the control versus dasatinib-treated explants, we used the GSEA (gene set enrichment analysis) software version 2.0.13 obtained from the Broad Institute (http://www.broad.mit.edu/gsea). We used the pathways defined by the Kyoto Encyclopedia of Genes and Genomes as the gene set in this study (REF). Gene set permutations were performed 1,000 times for each analysis. We used the nominal p-value and Normalized Enrichment Score obtained from GSEA to sort the pathways up and down regulated in the dasatinib-treated groups.

### Statistical analysis

An unpaired Student t-test was used to determine whether the means between control and dasatinib were significant at end of treatment (28 days). The differences were considered significant when the p value were <0.05. All error bars are represented as the SEM. A tumor growth index (TGI) was calculated (mean tumor/mean control x 100) and a cut-off point of 50% was used to dichotomize treatment effects into sensitive (TGI < 50%) and resistant (TGI ≥ 50%). All analyses were carried out by GraphPad Prism version 5.0c for Windows (GraphPad Software).

## Results

### Efficacy of dasatinib in CRC cell lines and CRC explants

**Fig 1:**
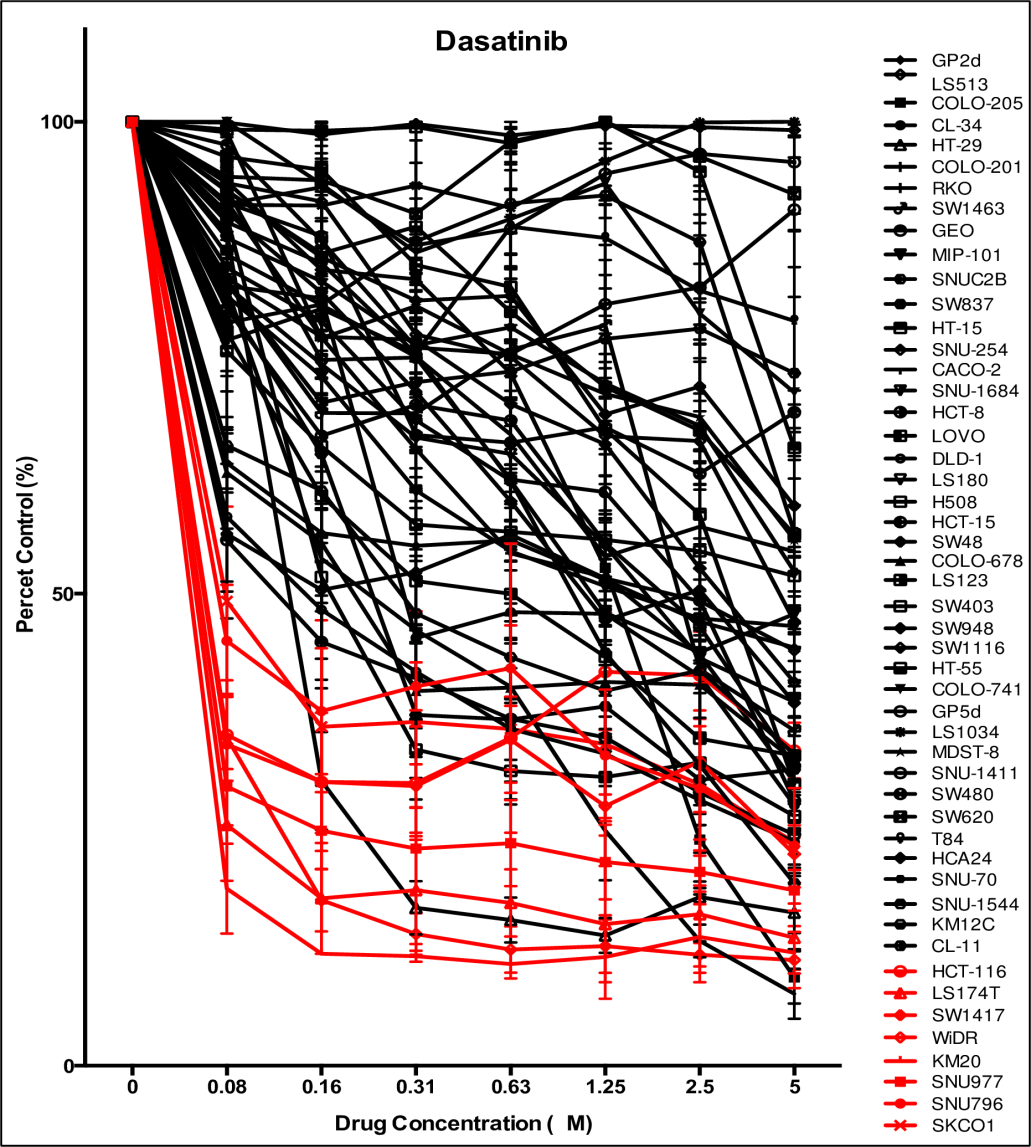
The effects of dasatinib on CRC cell lines *in vitro*. Fifty CRC cell lines were treated with dasatinib (dose 0.08–5 μmol/L) and proliferation was determined by an SRB assay. An IC_50_ ≤ 0.08 μM were deemed sensitive to dasatinib. The CRC cell lines KM20, LS174T, SNU-977, WiDR, HCT-116, SW1417, SNU-796, and SKCO1 demonstrated sensitivity to dasatinib.

**Fig 2:**
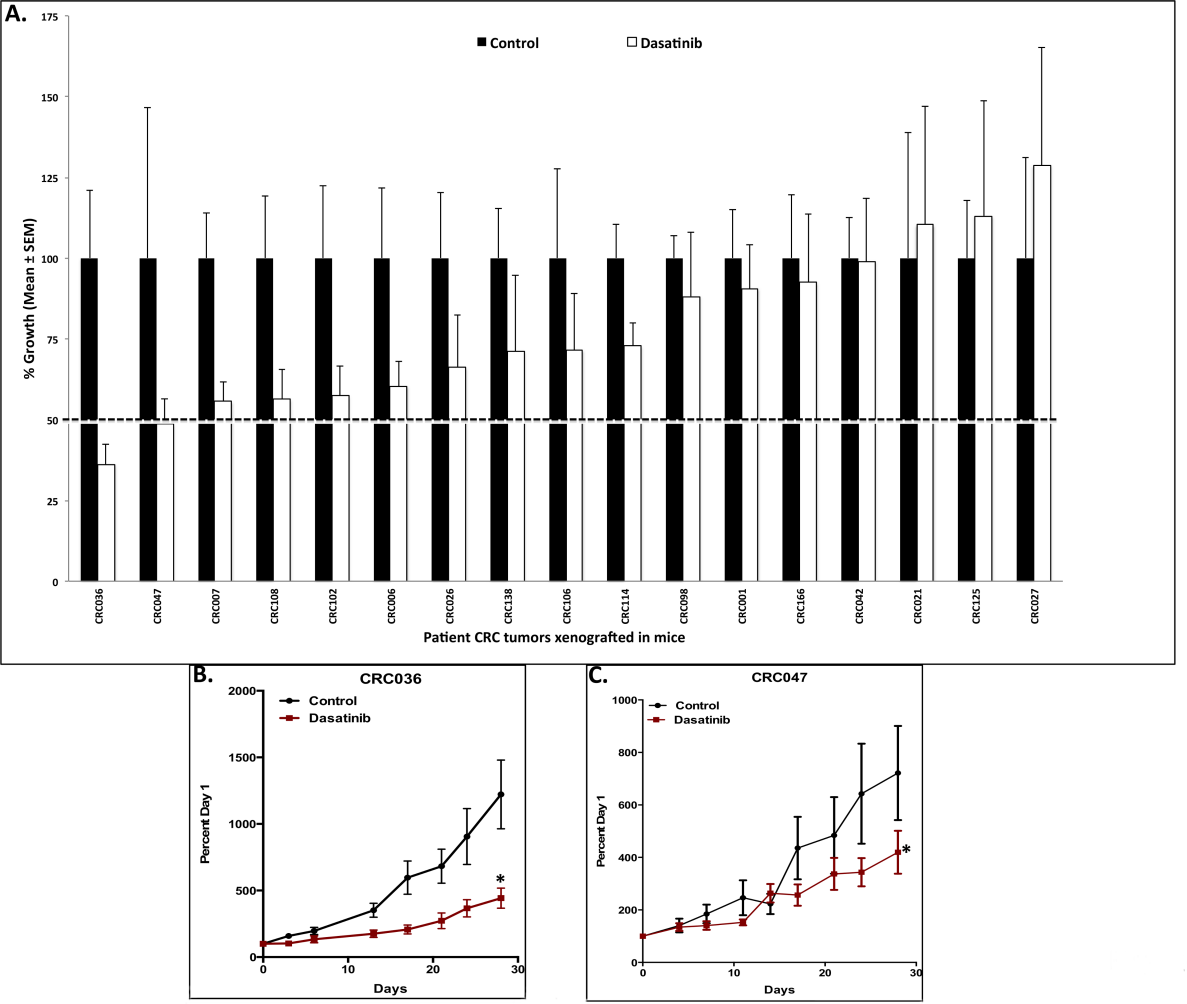
Efficacy of dasatinib in CRC PDX models. A) Seventeen colorectal cancer explants were treated with dasatinib and tumor size was evaluated twice per week. A TGI was calculated by relative tumor growth of treated mice divided by relative tumor growth of control mice x 100. CRC explants that exhibited a TGI < 50% were considered sensitive and TGI > 50% were considered resistant to dasatinib. B-C) Two explants (CRC036 and CRC047) demonstrated sensitivity to dasatinib. Columns, mean (n = 10 tumors per group); bars, SEM; and *, significance (P < 0.05) compared with control.

We first investigated the antiproliferative effects of dasatinib on 50 CRC cell lines. As demonstrated in Fig 1, a subset of CRC cells were sensitive to dasatinib, defined as an IC_50_ ≤ 0.08 μmol/L (dose range 0.08–5 μmol/L). A cut-off of 0.08 μmol/L was used to determine sensitivity in CRC cell lines considering this is the concentration that is achievable in humans. The CRC cell lines HCT116, LS174T, SW1417, WiDR, KM20, SKCO1, SNU-796, and SNU977 all reached an IC_50_ < 0.08 μM. All other cell lines were shown to be more resistant to dasatinib. Fig 1 summarizes the IC_50_ as well as the clinically relevant mutational analyses of the CRC cell lines. Next, we evaluated treatment effects on 17 unique CRC explants grown in a xenograft mouse model and treated with dasatinib 50 mg/kg/day, administered once-daily for 28 days. Table 1 displays the patient characteristics such as tumor type, stage of disease, previous treatment and common mutational analyses in CRC. Of the 17 CRC explants, CRC036 and CRC047 were shown to exhibit sensitivity (TGI < 50%) to dasatinib (Fig 2). The remaining 15 CRC explants displayed more treatment resistance to dasatinib.

**Table 1:** Patient characteristics.

Specimen ID	Colon or Rectal	Age at Consent	Primary or Metastatic	Previous Treatment	Stage	KRAS	PIK3CA	BRAF	TP53
CRC-001	Colon	69	Primary	FOLFOX plus bevacizumab	IV	Mut	WT	WT	WT
CRC-006	Colon	42	Primary	Capecitabine plus radiation, FOLFOX plus bevacizumab, Irinotecan plus cetuximab	IV	Mut	WT	WT	
CRC-007	Colon	47	Primary	None	II	Mut	WT	WT	Mut
CRC-021	Rectal	71	Primary	None	II	Mut	WT	WT	Mut
CRC-026	Colon	48	Metastatic	FOLFOX plus bevacizumab	IV	WT	WT	WT	WT
CRC-027	Colon	56	Metastatic	None	IV	Mut	WT	Wt	Mut
CRC-036	Rectal	42	Metastatic	Capecitabine plus radiation, FOLFOX plus bevacizumab, Irinotecan plus cetuximab	IV	Mut	WT	WT	WT
CRC-042	Rectal	73	Primary	FOLFIRI plus bevacizumab	II	Mut	WT	WT	Mut
CRC-047	Rectal	58	Primary	None	II	WT	WT	WT	
CRC-098	Colonc	50	Metastatic	None	IV	Mut	Mut	WT	Mut
CRC-102	Rectal	55	Metastatic	FOLFOX	IV	Mut	WT	WT	Mut
CRC-106	Colon	57	Metastatic	FOLFOX plus bevacizumab	IV	WT	WT	WT	WT
CRC-108	Colon	44	Metastatic	Capecitabine, oxaliplatin plus bevacizumab	IV	Mut	WT	WT	WT
CRC-114	Colon	70	Primary	None	III	WT	WT	WT	WT
CRC-125	Rectal	58	Metastatic	Unknown	IV	WT	WT	WT	Mut
CRC-138	Colon	78	Metastatic	None	IV	Mut	WT	WT	Mut
CRC-166	Rectal	41	Metastatic	FOLFOX plus bevacizumab	IV	WT	WT	WT	WT

### Cell cycle and apoptosis analysis of sensitive and resistant CRC cell lines treated with dasatinib

**Fig 3:**
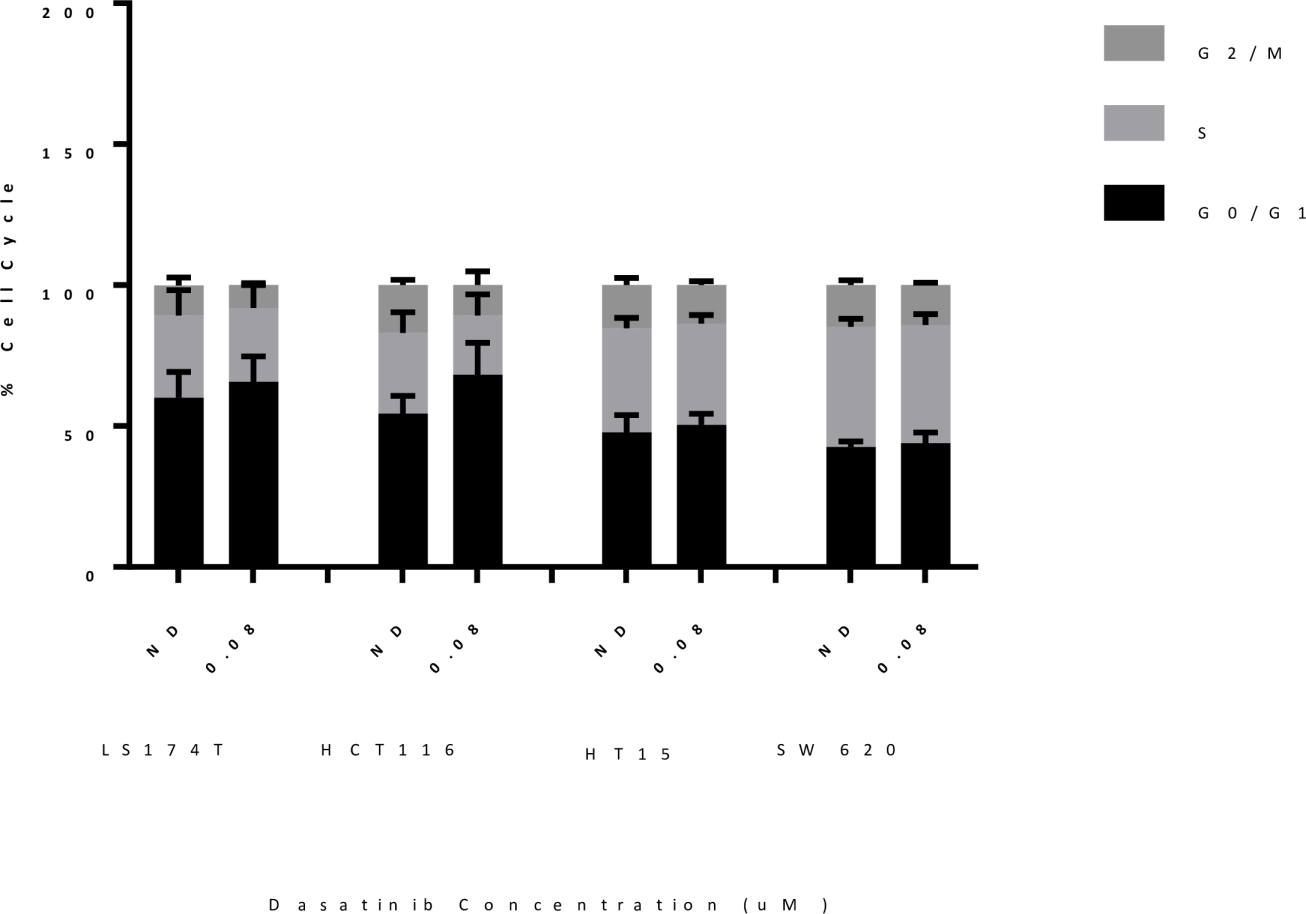
Cell cycle analysis of sensitive and resistant CRC cell lines. Sensitive CRC cell lines (HCT116 and LS174T) demonstrated an increase in G1 cell cycle arrest compared to resistant cell lines (HT15 and SW620) after treatment with dasatinib.

To examine the anti-proliferative mechanisms of Src inhibition, two sensitive (HCT116 and LS174T) and two resistant (HT15 and SW620) CRC cell lines were treated with dasatinib (0.08 μmol/L) and the proportion of cells cycling in G1, S and G_2_M were determined after 24 hours of treatment. As shown in Fig 3A, an elevation in G1 was observed in both the sensitive cell lines LS174T and HCT116. In contrast, an increase in G1 was not seen with dasatinib treatment in the resistant cell lines HT15 and SW620. In addition, we assessed whether dasatinib induces apoptosis after treatment; no increase in apoptosis was seen in the sensitive and resistant cell lines measured by caspase 3/7 assay (Fig 3B). These results demonstrate that the treatment effects of dasatinib on tumor growth are cytostatic and not cytotoxic.

### Investigation of Src activity after treatment with dasatinib in the CRC cell lines and explants

**Fig 4:**
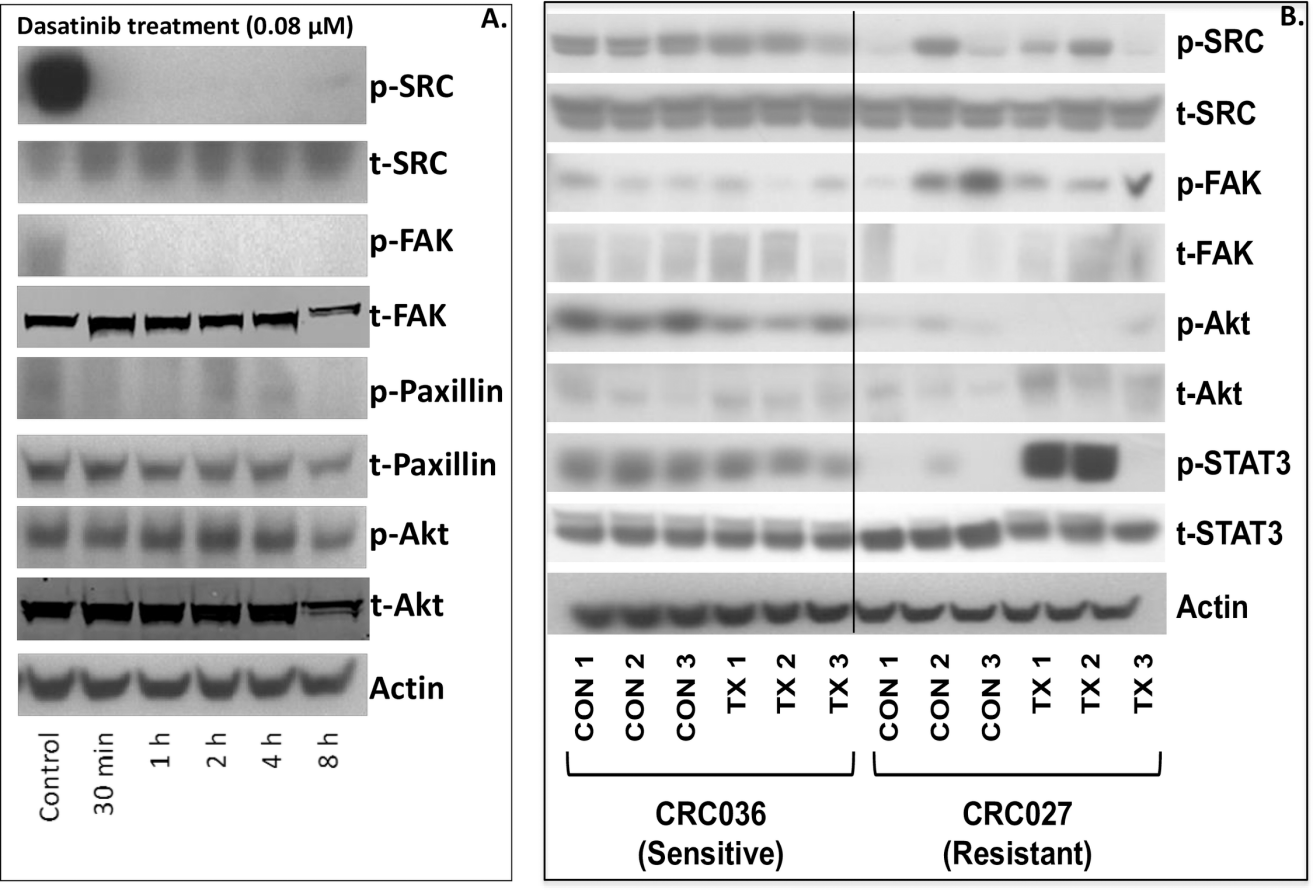
Pharmacodynamic effects of dasatinib on the Src pathway. A) Treatment with dasatinib (0.8 μmol/L) at 0.5h, 1h, 2h, 4h, and 8h significantly reduced the activation of Src, FAK and paxillin at all time points examined in the HCT116 sensitive CRC cell line when compared to control. B) A decrease in Src activity was seen in 1 out of 3 CRC explants treated with dasatinib in the sensitive (CRC036) and resistant CRC explant (CRC027) measured at end of study (day 28). However, in both cases FAK activity appeared to be increased.

We next evaluated the treatment effects of dasatinib on the Src signalling pathway on the sensitive HCT116 CRC cell line and CRC036 explant as well as on the resistant CRC explant CRC027. In the HCT116 CRC cell line we demonstrated a significant reduction in the activation of Src and the downstream kinases FAK and paxillin as early as 30 minutes of treatment (Fig 4A). This decrease was seen throughout the entire 8 hour time point examined. In addition, we observed a decrease in AKT activation after 8 hours of dasatinib treatment. In our CRC explants, a significant decrease in Src activity was seen in 1 out of 3 tumors treated with dasatinib in CRC036; however, the FAK activity level appeared to be increased (Fig 4B). Dasatinib treatment reduced the activation of AKT in all 3 tumors examined. Similar variability was observed in the resistant CRC027 explant.

### Src/FAK pathway is upregulated in CRC cell lines sensitive to dasatinib

**Fig 5:**
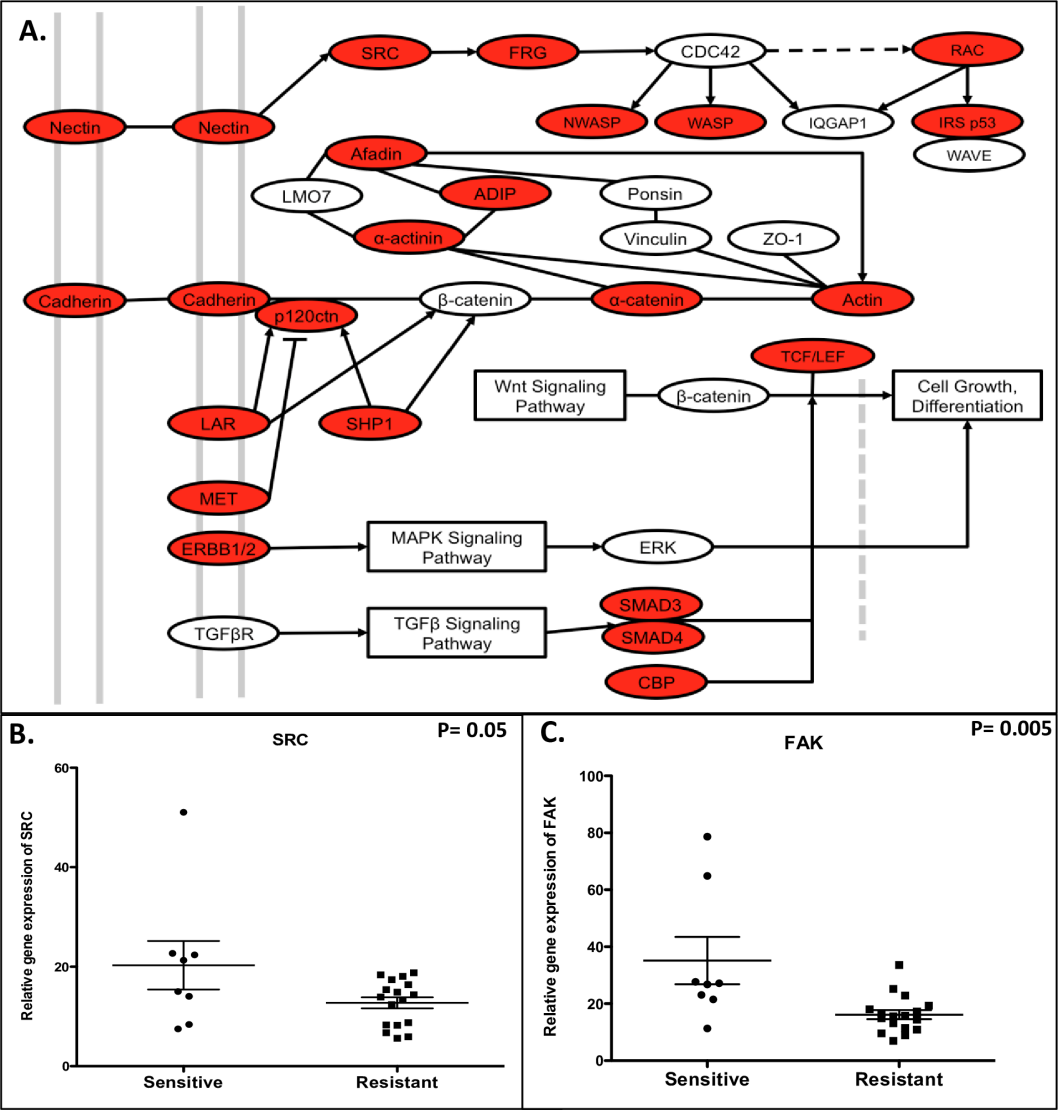
Evaluation of baseline pathway gene expression between sensitive and resistant CRC cell lines. A) Pathway analysis of sensitive and resistant gene expression and KEGG pathway diagram shows that adherens junction is a pathway enriched in sensitive CRC cell lines (red shows the genes that are increased in the sensitive cell lines and include Src and FAK. B) Src and C) FAK gene expression are significantly elevated in sensitive when compared to resistant CRC cell lines.

We performed a comprehensive pathway analysis via gene array on the 8 sensitive (0.08 μmol/L) and 18 most resistant CRC cell lines (Fig 1) to compare differences in pathways between the sensitive and resistant CRC cell lines. As shown in Fig 5A, one of the top pathways enriched in the sensitive cell lines when compared to the resistant cell lines was adherens junction. The genes included in this pathway are Src and FAK (shown in red are genes upregulated in sensitive compared to resistant). In addition, analysis of Src (Fig 5B) and FAK (Fig 5C) gene expression by RNA Seq show that both Src and FAK are significantly upregulated at basline in sensitive versus resistant CRC cell lines. These results suggest that dasatinib has greater anti-proliferative activity in CRC cell lines that exhibit higher levels of Src and FAK gene expression.

## Discussion

The importance of Src in tumor development, migration, and proliferation has been elucidated over the past century [22, 23]. Despite it being a common oncogenic aberrancy in a variety of solid tumors including CRC, targeting Src in the treatment of solid tumors has demonstrated mixed results. Based on previous preclinical data showing activity of Src inhibition in CRC cell lines, this study sought to investigate the efficacy of dasatinib, an ATP-site competitive, orally bioavailable inhibitor of Src family kinases, BCR-ABL, ephrin (EPH), c-Kit, and PDGFRβ, in the treatment of CRC cell lines and xenograft mouse models [19, 24].

We demonstrated that dasatinib showed anti-proliferative and antitumor effects in 8 out of 50 CRC cell lines and 1 out of 17 CRC explants, respectively. The IC_50_ was ≤ 0.08 μmol/L in 8 sensitive cell lines, which is a dose achievable in humans [25]. In those cell lines that demonstrated sensitivity to dasatinib, the relative pre-treatment gene expression of Src and FAK were significantly higher than the resistant cell lines based on Western Blot analyses, suggesting Src expression may offer a role as a predictive biomarker. Conversely, other preclinical studies have shown that the correlation of Src expression and tumor response to Src inhibition in the treatment of other tumor types including thyroid cancer cell lines is less clear, and p-FAK may be more predictive of response in thyroid cancer (Schweppe, 2009; Klutchko, 1998; Kraker, 2000).

The mechanism of Src inhibition occurs through cell cycle arrest during G1 phase as supported by our cell cycle arrest assay, consistent with other preclinical studies investigating other solid tumor types [26, 27]. Subset analysis of clinically relevant mutational status, such as KRAS, BRAF, and PIK3CA, did not show any correlation with efficacy. Anti-tumor effects in our PDX mouse model showed only modest results with dasatinib treatment. These results are consistent with our previously published *in vivo* and *in vitro* experiments investigating Saracatinib (AZD0530), a Src inhibitor, in the treatment of CRC cell lines and explants [28]. Zheng *et al* has also shown that Src siRNA knockdown decreased proliferation rate of the HCT-116 cell line similar to our results with dasatinib treatment [29].

The limited efficacy of Src inhibition in our study is consistent with previously published clinical studies using single-agent dasatinib [30–32]. Combinational approaches with dasatinib in addition to chemotherapy have shown some promise. In a phase 1b trial studying dasatinib in combination with cetuximab and FOLFOX in patients with metastatic CRC, Lieu *et al*. reported that 24% of patients achieved a partial response (PR) with a 17% PR rate in patients previously reported to be refractory to FOLFOX and cetuximab [33]. Results from another recently published phase 1 clinical trial using dasatinib plus capecitabine, oxaliplatin, and bevacizumab showed that patients with high expression of Src had a 75% overall response rate compared to 0% in low expression (p = 0.007) [34]. Based on the results from this study, a phase II trial investigating dasatinib in combination with chemotherapy is ongoing (NCT00920868).

Lack of efficacy of dasatinib may be due to multiple factors; including unselective properties as a multi-kinase inhibitor, lack of predictive biomarker data, variability of Src biology and role in CRC tumorigenesis, and efflux of dasatinib from CRC cells via activation of p-glycoprotein (p-gp), a member of the ABC transporter family (Ekblad 2010). There is also evidence that resistance to Src inhibition may be related to Src-induced reduction in immune responses (Abram, 2008; Sillaber, 2009).

Despite limited efficacy, this study suggests that increased Src expression may be a potential predictive biomarker to guide treatment with Src inhibition in patients with advanced CRC. In addition, preclinical data investigating combinational approaches has demonstrated that dasatinib sensitizes *KRAS* mutant CRC tumors to anti-EGFR therapy *in vitro* and *in vivo*, suggesting that there may be additional biomarker data yet to be fully elucidated to help drive future studies [35]. Similarly, Perez *et al* found that addition of dasatinib to fluoruracil and oxalplatin re-sensitize CRC tumors that expressed high levels of p-SRC [36] Biomarker driven data will likely determine the future of Src inhibitors in combination with chemotherapy for the treatment of solid tumors. Multiple Src inhibitors in combination with a variety of chemotherapies are currently being studied in phase II/III clinical trials in patients with solid tumors.

### Conclusion

Src plays an important role in tumor migration and proliferation, and is commonly found to be upregulated in CRC tumors. This has made Src inhibition an attractive target for treatment of CRC. Dasatinib, a Src kinase family inhibitor, showed anti-tumor activity in a subset of CRC cell lines and in one explant mouse model. The greatest effects were seen in cell lines that had elevated baseline levels of Src activity; however, the baseline level of Src activity in the explant models did not correlate with improved responses. The anti-tumor activity of single-agent Src inhibition appears to be limited, and the focus of Src inhibition in future studies should be limited to combinational approaches in the treatment of CRC.

## Declarations

### Ethics approval and consent to participate

Patient-derived colorectal adenocarcinoma tumor specimens were obtained from consenting patients at the University of Colorado Hospital in accordance with a protocol approved by the Colorado Multiple Institutional Review Board (08–0439).

### Consent for publication

Patients signed informed consent to participate in this research.

## Supporting information

S1 Table Summary of the IC_50_ as well as the clinically relevant mutational analyses of the CRC cell lines. (XLSX)

## Abbreviations

CRC: colorectal cancer
PDX: patient derived tumor xenograft
FAK: focal adhesion kinase
TGI: tumor growth inhibition
ATCC: American Type Culture Collection
ECACC: European Collection of Cell Cultures
KCLB: Korean Cell Line Bank
EGFR: epidermal growth factor receptor
RTK: receptor tyrosine kinase
p-gp: p-glycoprotein
HER2/neu: human epidermal growth factor receptor 2/Neu
PDGFR: platelet-derived growth factor receptor
FGFR: fibroblast growth factor receptor
HGF: hepatocyte growth factor
CSF-1R: colony stimulating factor-1 receptor
IGF-1R: insulin-like growth factor-1 receptor
c-Kit: stem cell factor receptor
ALL: acute lymphoblastic leukemia
CML: chronic myelogenous leukemia
